# SiLiF Neutron Counters to Monitor Nuclear Materials in the MICADO Project

**DOI:** 10.3390/s21082630

**Published:** 2021-04-08

**Authors:** Luigi Cosentino, Quentin Ducasse, Martina Giuffrida, Sergio Lo Meo, Fabio Longhitano, Carmelo Marchetta, Antonio Massara, Alfio Pappalardo, Giuseppe Passaro, Salvatore Russo, Paolo Finocchiaro

**Affiliations:** 1 INFN Laboratori Nazionali del Sud, 95123 Catania, Italy; cosentino@lns.infn.it (L.C.); giuffrida@lns.infn.it (M.G.); fabio.longhitano@lns.infn.it (F.L.); marchetta@lns.infn.it (C.M.); massara@lns.infn.it (A.M.); alfio.pappalardo@eli-np.ro (A.P.); passaro@lns.infn.it (G.P.); russos@lns.infn.it (S.R.); 2 CEA, DES, IRESNE, Nuclear Measurement Laboratory, Cadarache, F-13108 Saint-Paul-lez-Durance, France; Quentin.ducasse@cea.fr; 3 Centro Siciliano di Fisica Nucleare e Struttura della Materia, 95123 Catania, Italy; 4 ENEA Centro Ricerche, 40129 Bologna, Italy; sergio.lomeo@enea.it

**Keywords:** neutron detectors, radwaste management, radwaste monitoring

## Abstract

In the framework of the MICADO (Measurement and Instrumentation for Cleaning And Decommissioning Operations) European Union (EU) project, aimed at the full digitization of low- and intermediate-level radioactive waste management, a set of 32 solid state thermal neutron detectors named SiLiF has been built and characterized. MICADO encompasses a complete active and passive characterization of the radwaste drums with neutrons and gamma rays, followed by a longer-term monitoring phase. The SiLiF detectors are suitable for the monitoring of nuclear materials and can be used around radioactive waste drums possibly containing small quantities of actinides, as well as around spent fuel casks in interim storage or during transportation. Suitable polyethylene moderators can be exploited to better shape the detector response to the expected neutron spectrum, according to Monte Carlo simulations that were performed. These detectors were extensively tested with an AmBe neutron source, and the results show a quite uniform and reproducible behavior.

## 1. Introduction

In the framework of the MICADO (Measurement and Instrumentation for Cleaning And Decommissioning Operations) European Union (EU) project [1], whose goal is to establish new, fully digital assessment and management of low- and intermediate-level radioactive waste, we have produced and characterized a full set of radiation counters suitable for monitoring gamma rays and neutrons. In particular the goal of the Work Package 7 is to set up a granular radwaste monitoring system to be used during the final MICADO demonstration [2]. Indeed, the practice so far has been to store the radwaste drums following their initial characterization, the only monitoring consisting in an overall set of few ambient detectors and periodic manual checks done by operators. The proposed system for online real-time monitoring consists of an array of many radiation sensors to be deployed all around a number of radioactive waste drums, in order to collect counting-rate data in real time and to make them available to a software platform under development named DigiWaste. In order to be suitable for mass deployment these detectors have to be compact, reasonably inexpensive, robust, easy-to-use and reliable.

Continuous radiological monitoring of radwaste has to be based on the measurement of gamma and neutron radiation, since these are penetrating types of radiation easily detectable out of the drums [3]. This is the reason why the focus in MICADO was placed on the development of detectors for gamma rays and neutrons. The overall system must be modular, so that one can easily modify number and placement of the sensors around the drums, and it has to be scalable in order to make it possible to tailor it to small-, medium- and large-scale storage configurations without conceptual limitations [4,5,6].

In this paper we focus on the neutron detection, and describe the SiLiF thermal neutron counters that we developed. They are based on a solid state silicon detector coupled to a thin layer of a neutron converter material, which upon capture of a low energy neutron produces an alpha particle and a triton that can be detected in the silicon [6,7,8,9,10,11,12,13,14,15,16,17,18]. As compared to the well known thermal neutron detectors, based on ^3^He whose price has been increasing and whose availability has been decreasing in the last decades, the SiLiF technology allows to achieve comparable performances at about one order of magnitude lower prices [7].

A set of SiLiF detectors was built and characterized by means of an intense AmBe neutron source whose flux was simulated and measured in the relevant positions. As a final exercise, using the characterization results, we made an estimate of the detection sensitivity with respect to the possible presence of small amounts of ^240^Pu inside a waste drum.

## 2. Materials and Methods

The mainstream technology for thermal neutron detection so far has been based on ^3^He, that is an artificially produced gas with a 5330 b cross section for the following reaction.

n + ^3^He → ^3^H (0.191 MeV) + p (0.573 MeV) (1)

The main source of ^3^He is the nuclear weapons program of USA and Russia, as a byproduct of the ^3^H (tritium) supply which undergoes a nuclear decay into ^3^He with a 12.3 years half life. The shortage of ^3^He in the last decades, along with its price increase, has led to a wide research for alternative neutron detection technologies [7].

The operating principle of the SiLiF neutron detector, as a viable alternative to ^3^He tubes [7], is straightforward: following a thermal neutron capture by ^6^Li, the ^7^Li compound nucleus decays into an alpha particle (^4^He) and a triton (^3^H), emitted back to back with high energy according to the reaction

n + ^6^Li→ ^3^H (2.73 MeV) + ^4^He (2.05 MeV) (2)

Semiconductor detectors, e.g., silicon diodes, can be used in combination with a neutron reactive film, usually made of ^6^Li or ^10^B and called neutron converter. Such a film converts thermal neutrons into charged particles which are then detected by the silicon diode. Indeed ^6^Li and ^10^B have a quite high cross section at thermal neutron energy, respectively 940 and 3840 b, which decreases with the inverse of the neutron velocity. In such a scheme fast neutrons can also be detected by surrounding the detector with a suitable moderator box, typically made from polyethylene, which slows neutrons down to thermal energy. The use of ^6^Li was preferred to ^10^B because, following a neutron’s capture, it has a unique decay channel with no gamma rays emitted and a higher available kinetic energy, and it decays into lighter particles that are easier to detect [8,9,10,11,12,13]. A valid neutron count is registered whenever the signal produced by a SiLiF detector, which is proportional to the energy deposited into the diode, is higher than a predefined threshold.

With this technique, moderately inexpensive solid-state neutron detectors featuring good gamma/neutron discrimination can be built. Since ^6^Li is chemically very reactive we decided to employ ^6^LiF, a stable salt enriched at 95% in ^6^Li, that can be deposited by evaporation onto a suitable substrate. Instead of the direct deposition onto the silicon diode we chose to use independent detector and converter, which allows a better modularity and reconfigurability [14].

SiLiF detectors in different configurations (single, sandwich, double sandwich) with various converter thicknesses have already been tested in laboratory with moderated AmBe sources, and also tested with neutron beams and with spent fuel. The collected data were compared with Monte Carlo simulation results [15] and with efficiency calibration data taken with a certified neutron source at a metrology institute [16], showing a perfect mutual agreement. These detectors can be successfully employed for long-term online monitoring of neutron radiation, and could represent a viable ingredient for both safety and security in a nuclear waste storage facility. The most convenient counting threshold can be suitably chosen according to the desired gamma/neutron discrimination and to the type of waste (i.e., neutron and gamma spectra expected) to be monitored.

Such an alternative to ^3^He tubes has several advantages:

- ^6^LiF is much cheaper and more easily available than ^3^He;
- any solid state detector can in principle be used to detect the secondary particles;
- it is operated at low voltage, typically 30–50 V as opposed to ≈1000 V of a gas detector;
- the semiconductor and the neutron converter can be replaced independently in case of damage;
- a double-sided silicon diode can be used to double the neutron detection efficiency [17,18].

In the framework of MICADO we had to build 32 SiLiF detectors, to characterize them, and to check their reliability and repeatability versus appropriate simulations. As for the detection behavior we made use of GEANT4 [19], widely used in the nuclear and particle physics community. For the neutron source simulation we opted for the Monte Carlo code Fluka [20], widely used and better suited for the simulation of fluxes for radiation protection applications, and finally the MCNP [21] code was employed for the simulation of a radwaste drum.

### 2.1. The Detector

According to the study and results shown in [15,16,17,18] we decided to employ the double-sided silicon detector MSX09-300, a 3 cm × 3 cm diode 300 µm thick with a 0.3 µm metallization Al layer on each face, produced by Micron Semiconductor Ltd., Lancing, UK. We remark that in principle any silicon diode can be changed into a SiLiF by applying a suitable ^6^LiF converter, and that the resulting geometrical efficiency scales up with the sensitive area. A wider diode, e.g., 5 cm × 5 cm, would almost triplicate the geometrical efficiency, but sensibly increasing the price and decreasing the signal-to-noise ratio due to the larger capacitance. Therefore the chosen diode was a tradeoff between price and performance.

**Figure 1:**
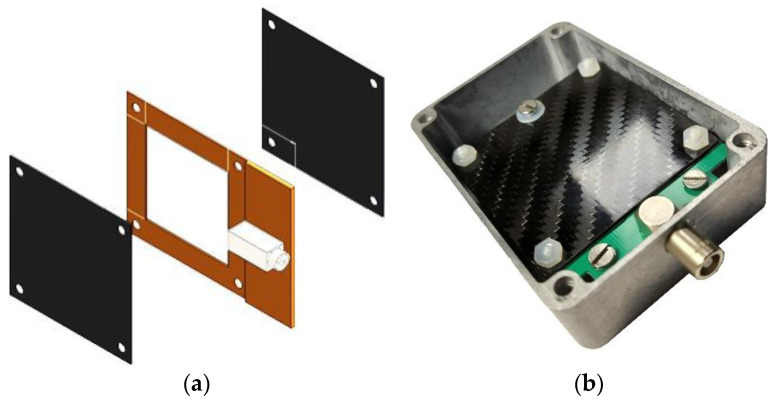
(**a**) The SiLiF detector arrangement as a double sided silicon diode sandwiched between two ^6^LiF layers deposited on carbon fiber substrates; (**b**) a real detector assembled inside its aluminum box.

**Figure 2:**
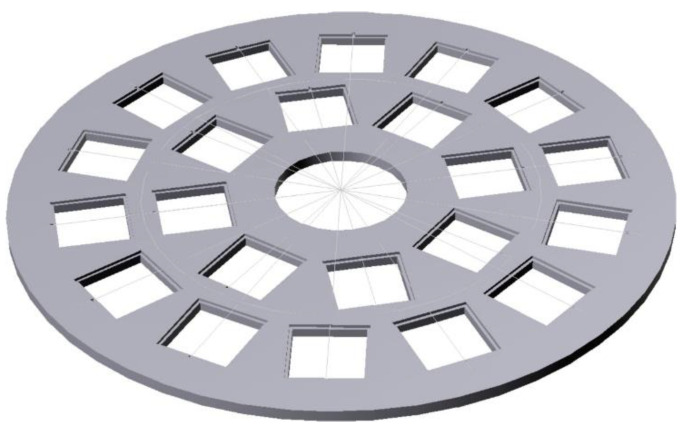
Figure **2.** Sketch of the rotating support explicitly constructed for the evaporation of the ^6^LiF converters in order to guarantee the uniformity of the deposition.

The detector configuration, shown in Figure 1, consists of the silicon diode sandwiched between two ^6^LiF converter layers deposited onto carbon fiber substrates. The evaporation of the converters was undertaken in three batches of 22 units, by means of a rotating support explicitly constructed for this purpose in order to guarantee the uniformity of the deposition (Figure 2). We remark that we used carbon fiber substrates from two different manufacturers, respectively 0.6 and 1 mm thick. We will show later that this has consequences.

**Figure 3:**
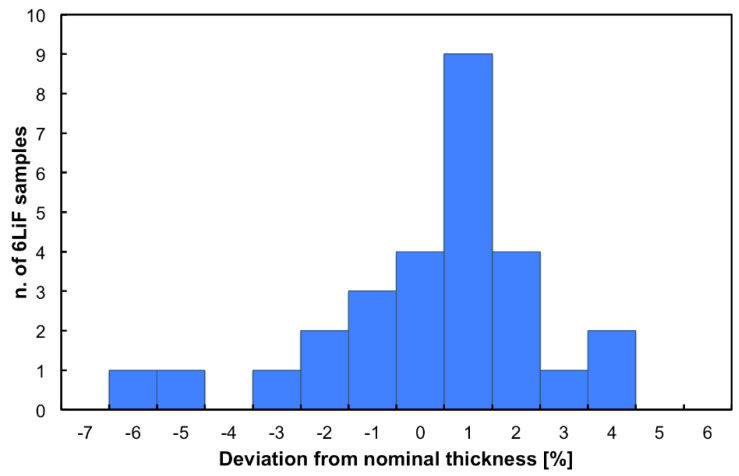
Distribution of the deviation of the effective areal density with respect to the nominal value (in percent) for 28 tested samples.

Following the results shown in Lo Meo et al. [15], the areal density chosen for the ^6^LiF layer was 4300 µg/cm^2^. For each evaporation session a few samples from the inner and outer rings were tested, 28 out of 66 in total, by comparing their weight before and after the deposition on a precision scale. This allowed us to calculate the amount of ^6^LiF effectively deposited. The results, summarized in Figure 3, indicate a slight systematic overdeposition of about 1% with respect to the nominal value, with a small dispersion of about 2% (standard deviation).

### 2.2. The Neutron Moderator

**Figure 4:**
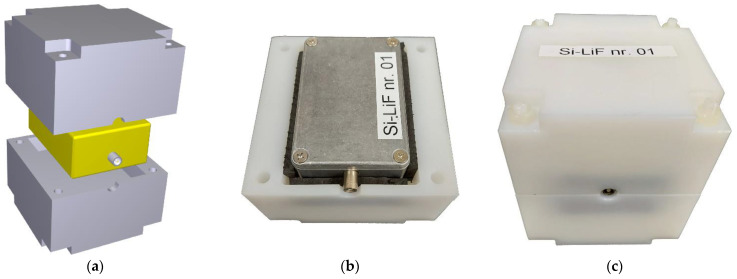
(**a**) 3D sketch of the SiLiF detector (in yellow) and its arrangement within the moderator (in grey); (**b**) a SiLiF hosted in a half moderator; (**c**) final assembling of a SiLiF.

The detection efficiency for thermal neutrons (i.e., 0.025 eV) is maximum, as the ^6^Li(n,t)^4^He cross section is 940 b and decreases as the inverse 1/v of the neutron velocity. In order to increase the detection efficiency for faster neutrons one makes use of a moderator, i.e., a material with low atomic number that can efficiently slow down the neutrons via multiple elastic collisions. The typical material used for this purpose is polyethylene (C_2_H_4_), which is a very convenient solid moderator, rich in hydrogen and easily machinable. The choice of the moderator shape and thickness is typically done by means of Monte Carlo simulations of the neutron transport, and is a tradeoff between size and moderation level to be achieved. Indeed, the thicker the moderator the more efficient moderation can be achieved, especially for high-energy neutrons that need more collisions to be thermalized. Unfortunately, if the lower energy neutrons undergo too many collisions, their chance to be captured by hydrogen via the ^1^H(n,γ)^2^H becomes increasingly high. Hence the choice of a thick but not too thick moderator. For our purposes we concentrated on the optimization of the moderator for neutrons with energy ranging from thermal to a few MeV, which is the typical spectral range one can expect out of a radwaste drum possibly containing fissile nuclear material. A moderator of 4 cm thickness, shown in Figure 4a, gave the best results and was replicated in 32 units. As the waste drums to be monitored could in some cases be embedded in a neutron moderating polyethylene matrix, we foresaw the possibility of using the detectors in the half-moderator configuration (Figure 4b), to avoid an overmoderation that would increase the chance for neutrons to be lost because they were captured in hydrogen. The detector in its standard configuration, with 10 × 10 × 10 cm^3^ size, is shown in Figure 4c.

**Figure 5:**
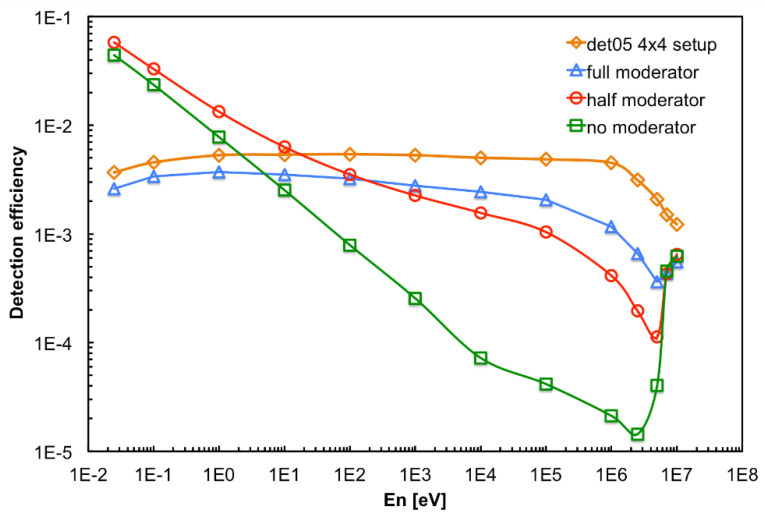
Simulated neutron detection efficiency in different moderator configurations (see the text for details).

The detector response to monoenergetic neutrons at 13 energies (0.025 eV, 0.1 eV, 1 eV, 10 eV, 100 eV, 1 keV, 10 keV, 100 keV, 1 MeV, 2.5 MeV, 5 MeV, 7 MeV, 10 MeV) was simulated in the full and half moderator configurations, along with the no-moderator one, by means of the GEANT4 code. The resulting detection efficiencies, plotted in Figure 5, clearly indicate that the full moderator provides a rather flat efficiency from thermal to 1 MeV. Conversely, as expected, in case of no moderator the detector is basically sensitive only to very slow neutrons. The half moderator configuration is a convenient solution when one expects neutrons already partially moderated. The lower detection efficiency below 1 eV observed for the full moderator, as compared to the other configurations, is due to a relatively important neutron capture by the hydrogen of the moderator. It is also clear that the moderator has little effect on the detection efficiency for neutrons at the highest energy (i.e., close to 10 MeV). The figure also shows the detection efficiency in a 4 × 4 square array configuration that will be described later.

### 2.3. The Neutron Source

**Figure 6:**
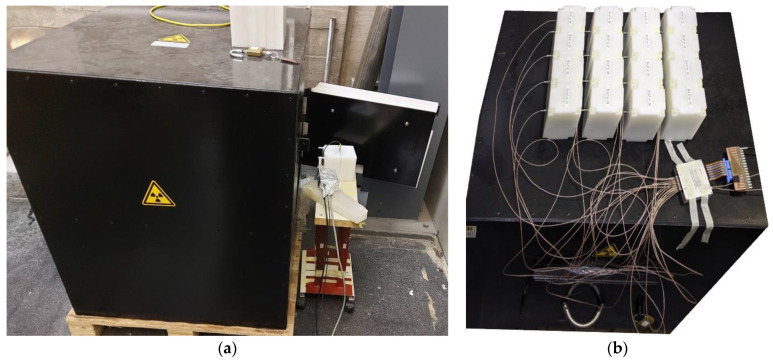
(**a**) The AmBe neutron source box with a SiLiF detector during a measurement in the front position. (**b**) The 4 × 4 array of SiLiF detectors during the measurement in the *top* position.

In order to test and characterize the detectors we made use of an intense AmBe source nominally emitting 2.2 × 10^6^ neutrons/s installed in an experimental hall at the INFN Laboratori Nazionali del Sud (LNS). Because of radiation protection requirements the source and its moderator are enclosed in a 95 × 75 × 85 cm^3^ iron box (Figure 6a). The source is surrounded by a first polyethylene case followed by 30 cm thick paraffin that slow down the high energy neutrons (up to 10 MeV) it emits. The outer 5 cm of the shielding are made from borated paraffin that stops the vast majority of the outgoing thermalized neutrons. Still a very small amount of fast neutrons can escape such a shield, as will be discussed later. We remark that this is a legacy source from the 60’s and the effective geometry of its moderator is only roughly known (i.e., the paraffin, whose actual density and uniformity are not exactly known). Due to its huge activity (≈34 GBq of 59 KeV gamma rays from ^241^Am), modifying the source arrangement was not allowed for radiation protection reasons, and only a restricted set of operations was possible. However, such a configuration, heavily disturbed by the surrounding material assembly, gives rise to multiple neutron scattering and to a huge abundance of gamma rays. This makes the source somehow resembling a radwaste drum and, therefore, constitutes a good exercise to test and characterize the detectors.

**Figure 7:**
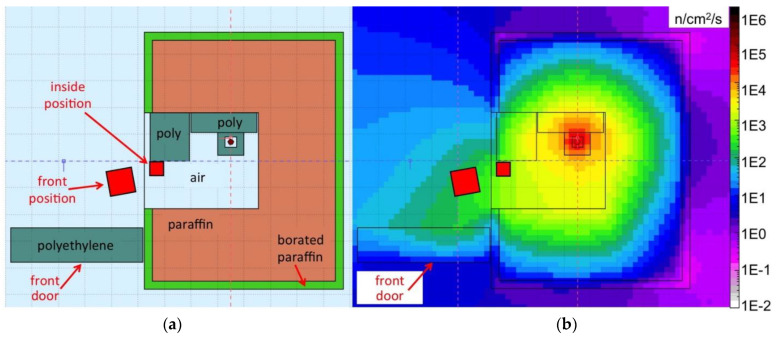
(**a**) Drawing of the top view of the section at 30 cm height of the neutron source box; the source position is indicated by a small dark circle, the red squares indicate the *front* and *inside* positions where the measurements were done; (**b**) the corresponding neutron flux obtained by means of a simulation with the Fluka code.

**Figure 8:**
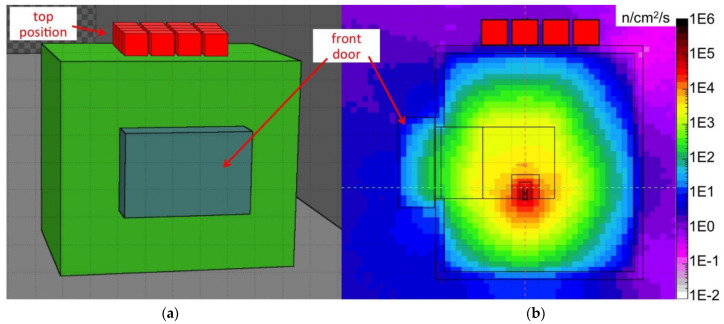
(**a**) Drawing of the 3D side view of the neutron source box, the red box indicates the *top* position; (**b**) the corresponding neutron flux obtained by means of a simulation with the Fluka code.

**Figure 9:**
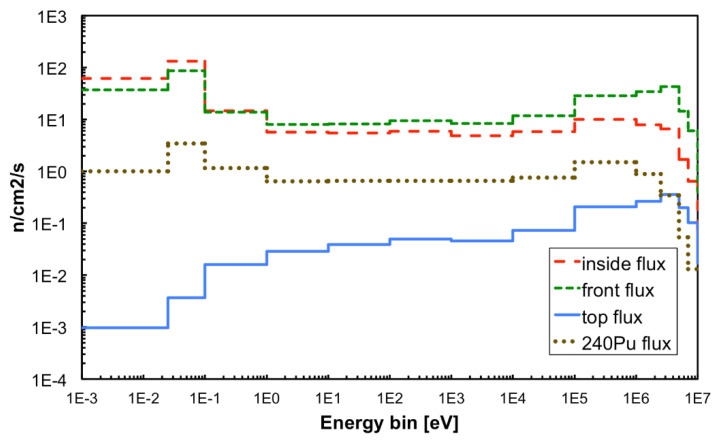
Neutron flux simulated in the three measurement positions with Fluka, and with MCNP on the detectors in the radwaste package exercise with ^240^Pu (see the Discussion section).

The source setup was simulated with Fluka [20], thus producing a 3D map of the expected neutron flux inside and around the box, as shown in Figure 7 and Figure 8. In addition, the expected neutron energy spectrum was simulated in three positions, namely *front*, *inside* and *top* highlighted with red boxes in the figures, where the SiLiF measurements were done. The three simulated spectra are plotted in Figure 9 (the *top* flux in the figure refers to det05 in the 4 × 4 array of Figure 6b, as explained later).

The total neutron flux expected in the *front* position is around 310 n/cm^2^/s, in the *inside* position it is expected around 263 n/cm^2^/s. The maximum flux expected in the *top* position, that is heavily shielded, is around 1.9 n/cm^2^/s.

## 3. Results

### 3.1. Measurements in the Front Position

**Figure 10:**
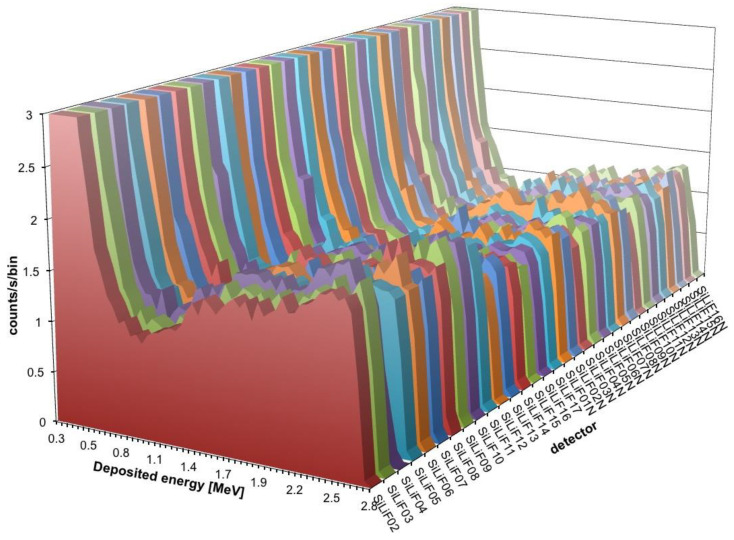
The characteristic spectrum shape, measured for all the SiLiF detectors in the front position, is due to the superposition of the triton (2.73 MeV) and alpha (2.05 MeV) contribution smeared down by the effect of the emission from different depths/angles in the ^6^LiF converter. The energy bin size is 47 keV.

The first set of measurements that was performed was a preliminary check that the detectors were operational and behaved as expected. Each detector in turn, inside its moderator, was placed on a small cart and positioned in front of the source just out of the box with the open door (Figure 6a). The detector was biased at 50 V, in order to operate in full depletion regime and be sensitive on both faces as required. The charge preamplifier employed was an ORTEC 142B, which has a nominal gain of 25 mV/MeV, connected to an ORTEC 672 spectroscopy amplifier whose output was sent to an Amptek MCA8000A multichannel analyzer. The resulting spectrum, as expected, reaches up to 2.73 MeV which is the maximum kinetic energy of the tritons when emitted perpendicularly from the surface of the ^6^LiF layer. The emission from deeper positions and/or with different angles gives rise to a lower energy deposition in the silicon, hence a low energy tail in the spectrum. The same applies to the alpha emission whose maximum energy is 2.05 MeV. The superposition of the triton and alpha deposited energy, plus the effect of the emission from different depths/angles, produces a characteristic spectrum shape, as can be observed in Figure 10 that also proves that all the detectors were actually working properly. The detector naming scheme in the figure is related to the original numbering from the manufacturer. The “N” suffix refers to a different (new) batch of detectors which has a higher depletion voltage.

### 3.2. Gamma/Neutron Discrimination

One of the main requirements for a neutron detector is the insensitivity to gamma rays. Actually, it is not possible to obtain such a perfect behavior, therefore one usually refers to the gamma/neutron discrimination e.g., the probability to detect a gamma ray and misinterpret it as a neutron. A γ/n count rate ratio typically accepted as quite good, with a detector in a mixed flux of neutrons and 1.17 and 1.33 MeV gamma rays from ^60^Co, is about 10^−4^ [22,23]. Profiting by the independent assembly of converter and diode we were able to simply evaluate γ/n in two steps.

**Figure 11:**
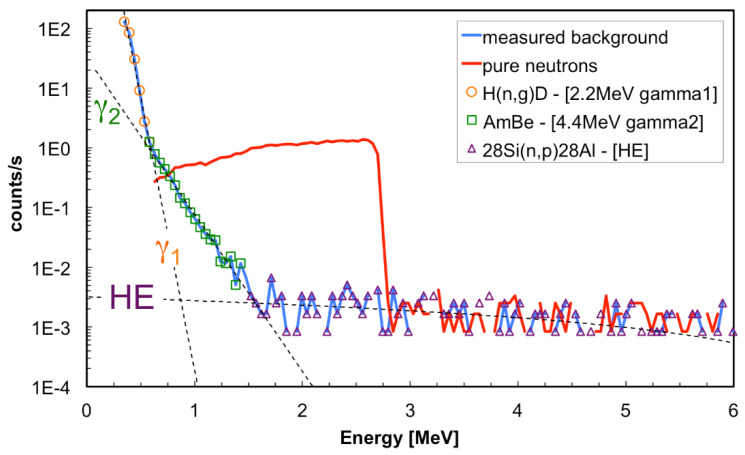
The background-subtracted neutron spectrum as compared to the background. Also highlighted are three different contributions to the background (see text).

First, we measured a normal spectrum in the *front* position then, after reversing the two carbon fiber substrates upside down, we repeated the measurement. Obviously, when measuring with the reversed converters no triton or alpha can reach the silicon diode, so that no detected event can be related to the ^6^Li(n,t)^4^He reaction and this is to be considered as background, even though there will be some additional consideration of it in the following. The background measurement was then subtracted from the real measurement, and the resulting spectrum is reported in Figure 11 as *pure neutron* spectrum, along with the measured background.

By observing the measured background one sees three contributions with clearly different behavior (we remark that no full energy gamma deposit is expected to occur in such a thin silicon detector):

- The first one, a decreasing exponential extrapolated in the figure up to 1 MeV, is mainly due to the abundant 2.2 MeV gamma rays produced by the neutron capture on hydrogen in the moderator box. We name it here “*gamma1*” contribution.
- The second one, a decreasing exponential extending up to 1.5 MeV and extrapolated in the figure up to 2 MeV, is due to the gamma rays produced by the AmBe source itself. Indeed, whenever an alpha particle from the americium reacts with the beryllium, this decays by emitting one neutron and one gamma ray, with the gamma energy between 3.4 and 4.4 MeV. We estimated that roughly the same number of such gamma rays and neutrons hit the detector in the *front* measurement position. Part of this contribution is also due to the elastic scattering of neutrons on silicon, with cross section of a few barn, and maximum silicon recoil kinetic energy, from 10 MeV neutrons, of about 1.33 MeV. We name it here “*gamma2*” contribution.
- The third one, with an almost constant linear trend toward high energy (“*HE*” contribution), is due to the ^28^Si(n,p)^28^Al reaction whose threshold is around 6 MeV and which has several resonances and cross section around 300 b. In this case both the proton and the recoiling ^28^Al deposit kinetic energy in the detector.

**Figure 12:**
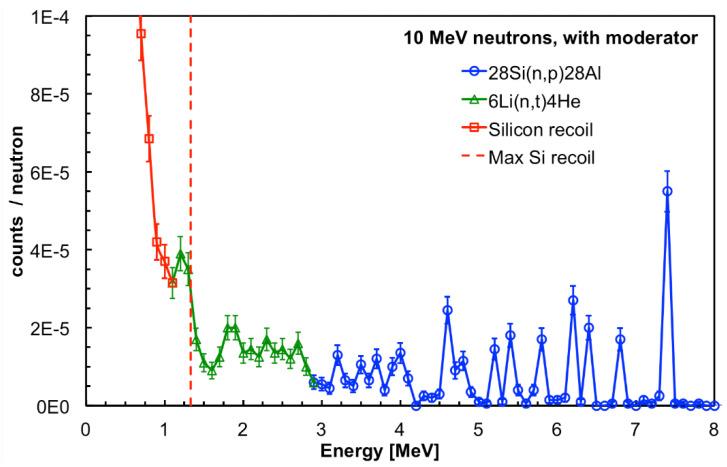
GEANT4 simulation of the SiLiF response to 10 MeV neutrons with the moderator surrounding the detector (see text).

**Figure 13:**
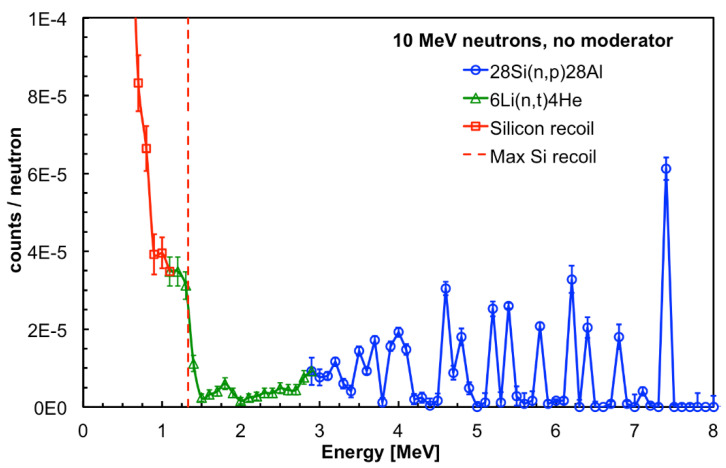
GEANT4 simulation of the SiLiF response to 10 MeV neutrons without the moderator surrounding the detector (see text).

The GEANT4 simulation of the SiLiF response to 10 MeV neutrons is shown in Figure 12 and Figure 13, respectively, with and without the moderator surrounding the detector. In both figures one can clearly see several structures above 3 MeV corresponding to resonances in the cross section of the ^28^Si(n,p)^28^Al reaction for which one measures the kinetic energy of the ^28^Al and of the proton, while the gamma ray from the ^28^Al de-excitation goes undetected. This confirms that 4 cm of polyethylene is not enough to efficiently thermalize 10 MeV neutrons, therefore a fraction of these neutrons retain a kinetic energy high enough for a nuclear interaction with the silicon nuclei. However, when the moderator is present there is still a contribution from the ^6^Li(n,t)^4^He reaction, visible in the 1 to 3 MeV range in Figure 12. Moreover, both figures show the low energy contribution below 1.33 MeV due to the recoil of silicon in the ^28^Si(n,n)^28^Si elastic scattering.

As a consequence one can make the following points:

- The counts above 2.73 MeV, i.e., the *HE* contribution, have to be ascribed to neutrons, even though not interacting with the ^6^LiF converter, and have to be considered in the detection efficiency evaluation.
- Fitting the two decreasing exponentials of Figure 11 can provide a realistic estimate of the gamma contribution in the measured neutron counts.

**Figure 14:**
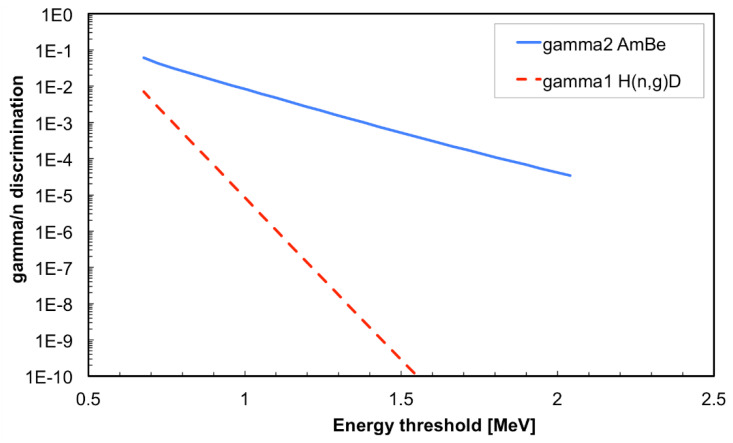
The γ/n ratio as a function of the chosen energy threshold, with respect to the *gamma1* and *gamma2* cases (see text).

Indeed, we calculated the ratio of the integrals of *gamma1* and *gamma2* to the integral of the *pure neutron* SiLiF spectrum for different energy thresholds. The resulting plot, shown in Figure 14, indicates the γ/n ratio as a function of the chosen energy threshold. Therefore, in cases where one only expects lower energy gamma rays (i.e., roughly below 2.2 MeV) a threshold at 1 MeV provides γ/n ≈ 10^−6^, and a threshold at 1.5 MeV γ/n ≈ 10^−10^, that is a very good discrimination.

If one expects much higher energy gamma rays, generally produced by the presence of alpha emitters and light elements like Li or Be, e.g., what happens with AmBe, a threshold at 1.5 MeV is recommended with γ/n ≈ 10^−4^. We recall that in the real data the *gamma2* region will also include a contribution of silicon recoil events produced by neutrons.

### 3.3. Intrinsic Detection Efficiency Measurement

**Figure 15:**
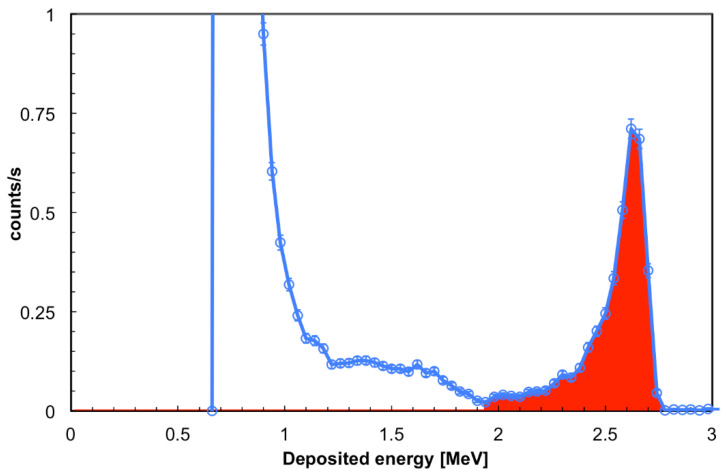
The deposited energy spectrum collected in the *inside* position with the reference detector. The colored area starts from the alpha endpoint, and contains 97% of the detected tritons (see text).

In order to determine the thermal neutron intrinsic detection efficiency of each SiLiF detector we needed to know the number of impinging neutrons in a given position, so that we could calculate it as the ratio of the number of neutrons detected to the impinging ones. This was achieved by making use of a reference detector which had been previously calibrated at a metrology institute in a certified thermal neutron field [16]. The reference detector is a SiLiF as well, with only a single layer of ^6^LiF converter 1.6 µm thick (410 µg/cm^2^). At first we measured the counting rate in the *inside* position with the reference detector, then we repeated the same measurement with each SiLiF detector. For this measurement all the detectors were used without their moderator, so that they were basically sensitive only to the thermal neutrons inside the big moderator box. The spectrum obtained with the reference detector is shown in Figure 15. The colored area extends upwards from the valley corresponding to the alpha endpoint, and contains 97% of the detected tritons. The detection efficiency under these conditions is 0.41% ± 0.001% [16], which implies that the impinging flux at the *inside* position was 107 ± 1.6 n/s/cm^2^ to be compared with an expected thermal flux of roughly 130 n/s/cm^2^ from the simulation. We remark that the alpha endpoint is slightly shifted to lower energy (see the red area in Figure 15) because of the energy loss in the thin air and aluminum dead layers between the converter and the silicon diode.

**Figure 16:**
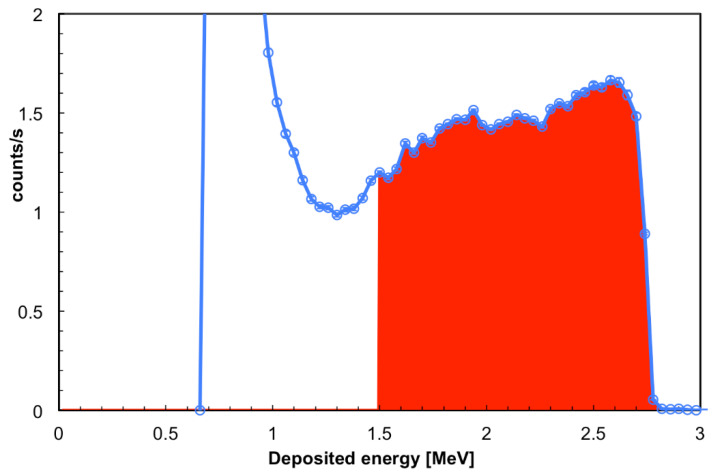
The typical deposited energy spectrum collected in the *inside* position with the SiLiF detectors under test. The colored area represents the number of detected neutrons for the chosen 1.5 MeV energy threshold, and was normalized to the impinging flux to provide the detection efficiency for the chosen threshold.

**Figure 17:**
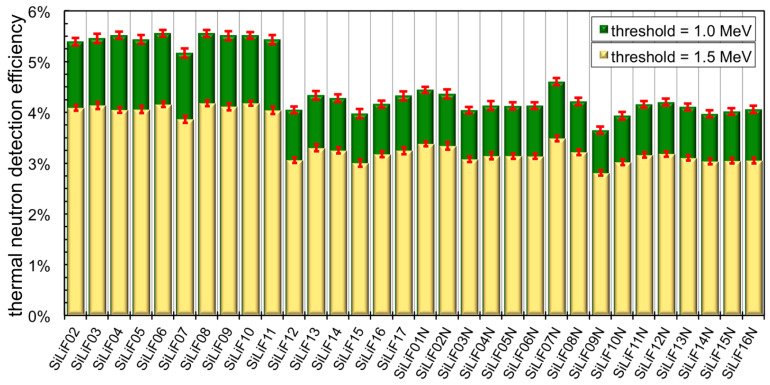
The intrinsic detection efficiency measured for the 32 SiLiF detectors in the *inside* configuration, with nominal energy thresholds of 1 and 1.5 MeV.

The typical spectrum measured with the SiLiF detectors under test at the *inside* position is shown in Figure 16. The colored area was normalized to the impinging flux and provides the detection efficiency for the 1.5 MeV energy threshold. The detection efficiency was evaluated for all the 32 detectors, with two nominal energy thresholds at 1 and 1.5 MeV, and the results are summarized in Figure 17.

We were surprised to discover that there were two distinct groups of detectors with different efficiency: detectors 02–11 (Figure 17) have a higher thermal neutron efficiency than the others. At first we supposed that this behavior was due to a different batch of silicon diodes, but this was excluded by swapping the converters between two different SiLiF detectors. We were forced to conclude that the difference was in the converter, even though the amount of ^6^LiF was the same.

**Figure 18:**
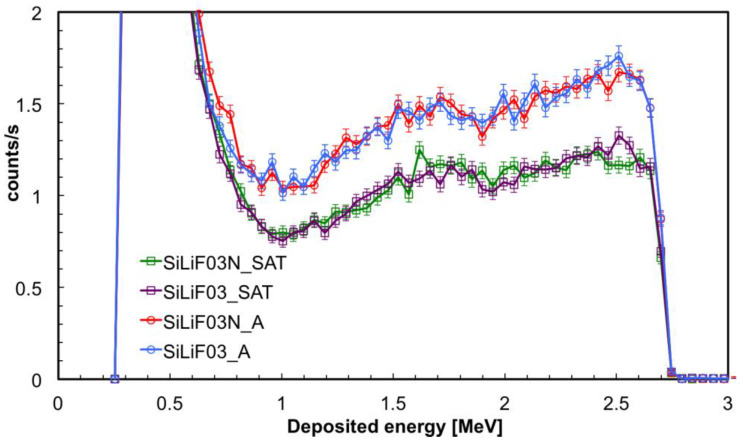
Deposited energy spectra obtained with the detectors SiLiF03 and SiLiF03N, belonging to two different silicon batches in the *inside* position. The detectors were coupled with ^6^LiF converters deposited onto carbon fiber substrates of the A and SAT types (coming from different manufacturers) which in a second run were swapped.

Then we realized that the carbon fiber had come from two different manufacturers, and discovered that the second one contains a resin with an unspecified amount of boron in it. Such an amount of boron captures a fraction of the impinging neutrons, thus reducing the thermal flux effectively reaching the ^6^LiF converter and in the end reduces the detection efficiency. To prove this we reported in Figure 18 the deposited energy spectra obtained with the detectors SiLiF03 and SiLiF03N, belonging to two different silicon batches. The detectors were coupled with ^6^LiF converters deposited onto carbon fiber substrates of the A and SAT types (coming from different manufacturers) which in a second run were swapped.

### 3.4. Test with Fast Neutrons

**Figure 19:**
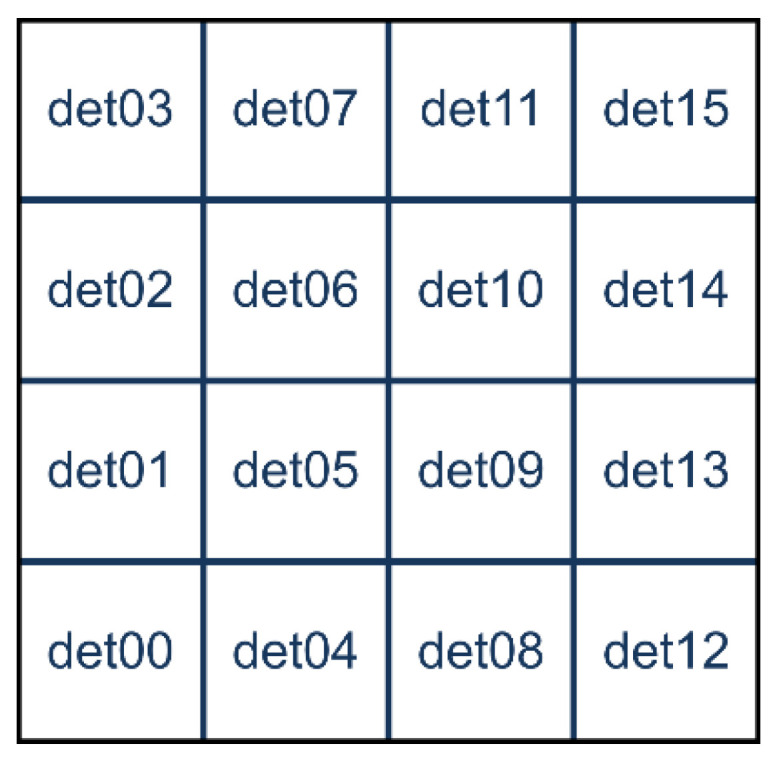
The 4 × 4 arrangement and numbering of the detector positions for the test with fast neutrons.

**Figure 20:**
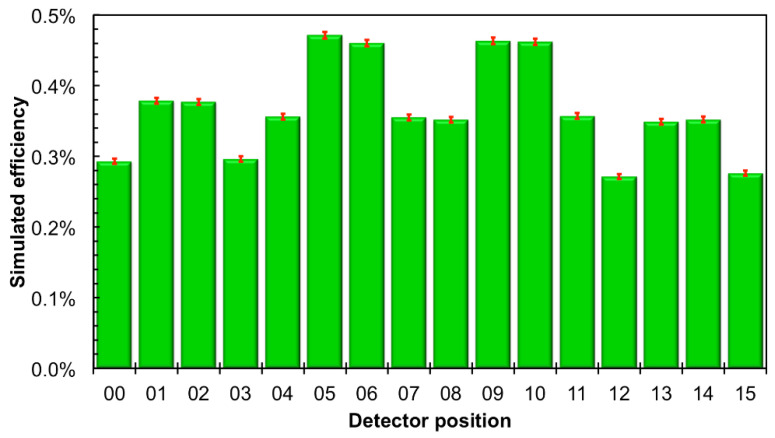
Behavior of the simulated average neutron detection efficiency between thermal and 2.5 MeV in the *top* position. The efficiency modulation due to geometrical effects is clearly visible. The error bars indicate only the statistical uncertainty.

A final test was performed using simultaneously 16 detectors aimed at measuring a low intensity flux of fast neutrons. To this purpose the detectors were arranged in a square 4 × 4 array in the *top* position upon the neutron source box. The efficiency of such a configuration, as resulting from a GEANT4 simulation, is flatter than in the single detector configuration. This is due to an improvement of the moderation of fast neutrons due to the contribution of neighboring moderators, as shown in Figure 5 for the detector in position 05. In Figure 19 we show the arrangement and numbering of the detectors, whereas in Figure 20 the behavior of the expected average detection efficiency between thermal and 2.5 MeV neutrons is reported. The efficiency modulation due to geometrical effects is clearly visible: the central detectors (05, 06, 09, 10) are the most efficient, the peripheral ones are less efficient (01, 02, 04, 07, 08, 11, 13, 14), and the corner ones are the least efficient (00, 03, 12, 15).

**Figure 21:**
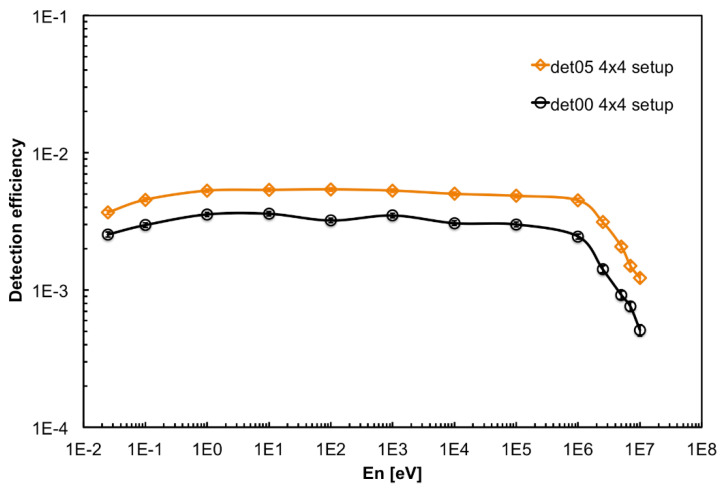
Comparison between a corner (det00) and a central (det05) detector simulated efficiency. The overall shapes are similar, the difference being essentially a scale factor enhancement for the central detector due to a geometrical effect of the neighboring moderators.

**Figure 22:**
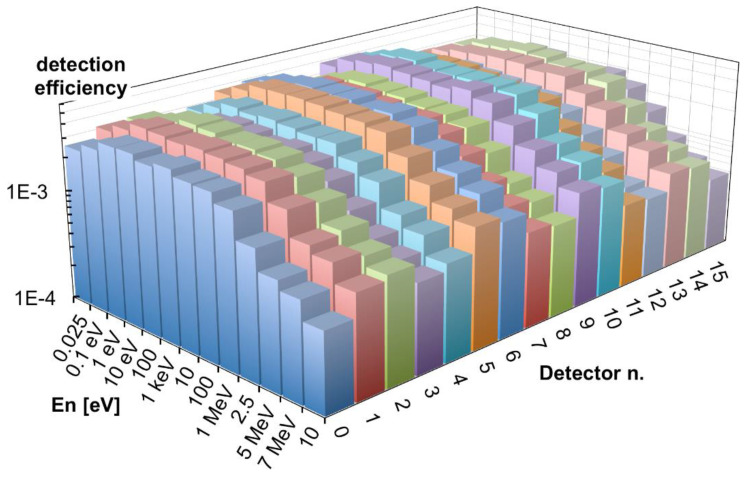
The simulated detection efficiency as a function of the impinging neutron energy for the 16 detectors in the 4 × 4 array configuration in the top position.

Moreover, a slight left-to-right decrease can be appreciated in Figure 20, due to the slightly asymmetric position of each silicon diode inside its box (Figure 1). A comparison between a corner and a central detector efficiency at various energies is shown as an example in Figure 21. In Figure 22 we show the simulated detection efficiency as a function of the impinging neutron energy for the 16 detectors in the 4 × 4 array arrangement.

**Figure 23:**
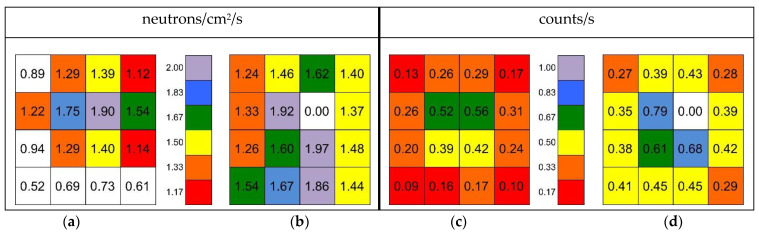
(**a**) Total neutron flux simulated in the *top* position in correspondence of the 4 × 4 SiLiF array; (**b**) The measured total neutron flux with the 4 × 4 array. (**c**) The simulated neutron counting rates. (**d**) The measured neutron counting rates.

The simulated neutron flux in the *top* position has a main contribution between 100 keV and 5 MeV (Figure 9), due to the presence of 5 cm borated paraffin in the outer layer of the source box which captures most of the low-energy neutrons. Its total value of 1.9 n/s/cm^2^ resulted in reasonable agreement with a previous direct measurement done by means of a portable neutron spectrometer Radeye PX/WENDI [24], which had provided a value of 2.09 n/s/cm^2^. By combining the simulated flux with the simulated detector efficiency we estimated the expected counting rate. Table 1 summarizes the comparison between measured and simulated counting rates in the *front* and *inside* positions. The neutron counts measured with the 4 × 4 array, corrected for the simulated efficiency and for the intrinsic detection efficiency (Figure 17), provided the total neutron flux for each detector. In Figure 23 we compare the (a) simulated and (b) evaluated fluxes, and then we compare the (c) simulated and (d) measured counting rates. Unfortunately, due to a broken channel in the electronics, the detector in position det10 did not provide useful data.

## 4. Discussion

In the previous sections we have shown that the SiLiF detectors can fruitfully be employed to detect neutrons, in a rather wide energy range and with a very good gamma rejection, in particular considering that typical commercial neutron detectors claim γ/n ≈ 10^−5^ with respect to the ^137^Cs gamma rays of 662 keV. This value is to be compared with the γ/n ≈ 10^−6^ of SiLiF with respect to 2.2 MeV gamma rays and the threshold at 1 MeV, or even better with the γ/n ≈ 10^−10^ if using a 1.5 MeV threshold. We have also seen that SiLiF detectors can provide realistic measurements of the total or thermal neutron flux, respectively, when used with or without moderator. The comparisons reported in Table 1 and Figure 23 have to be considered as being in good agreement. Indeed, due to the only roughly known features of the source moderator box, we would have been satisfied by an agreement between measurements and simulations within one order of magnitude. Quite surprisingly, the agreement came out much better, thus suggesting that the assumptions on the source geometry were realistic.

The agreement between the flux measured with the SiLiF detectors and with the portable neutron spectrometer in the *top* position is quite reasonable as well. It is also interesting to note that the measured flux (Figure 23b) suggests a spatially wider flux than indicated by the simulation (Figure 23a).

**Figure 24:**
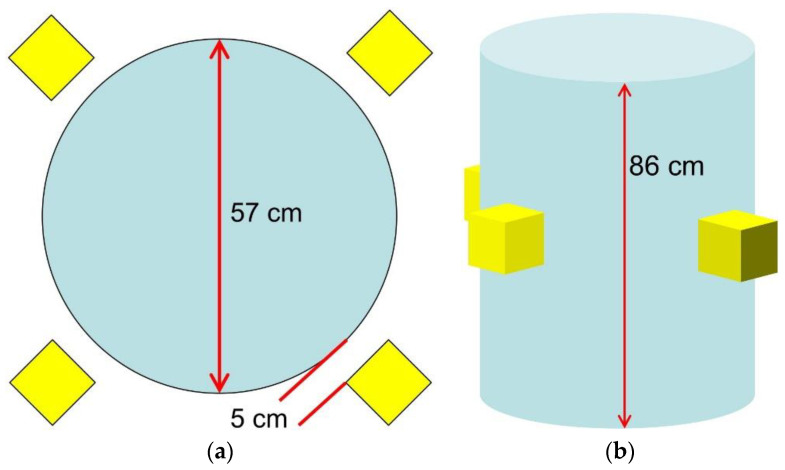
Sketch of the simulated radwaste package along with four SiLiF detectors around it. (**a**) Top view; (**b**) perspective view.

These results prove that SiLiF detectors can successfully be employed in the MICADO project in order to demonstrate the reliability of neutron monitoring in a medium-/long-term storage of radioactive waste drums. An interesting exercise has been done in such a framework, to investigate the possibility of detecting small quantities of actinides in low/intermediate level waste drums. A possible radioactive waste package (RWP) assembly was considered, enclosed in a standard 220 l drum (86 cm height, 57 cm diameter) with a steel/polyethylene matrix (67/33% in mass), in order to estimate the SiLiF sensitivity. The drum, sketched in Figure 24 along with four SiLiF detectors around it, was supposed to contain 100 g of ^240^Pu, which is an isotope typically present whenever there is plutonium. We calculated the rate of prompt-fission neutrons produced by such an amount according to the data in Table 2. Then we made a rough estimate of the total expected rate of neutrons impinging on the four silicon detectors, considering a uniform output flux from the cylinder surface, assuming a 10% neutron absorption inside the drum, and fully thermalized neutrons reaching the 9 cm^2 6^LiF converter. In Table 3 we listed the relevant numbers for the calculation, and the resulting neutron counting rate in the set of four detectors is about 4.5 counts/s, i.e., about 1.1 counts/s in each detector.

**Figure 25:**
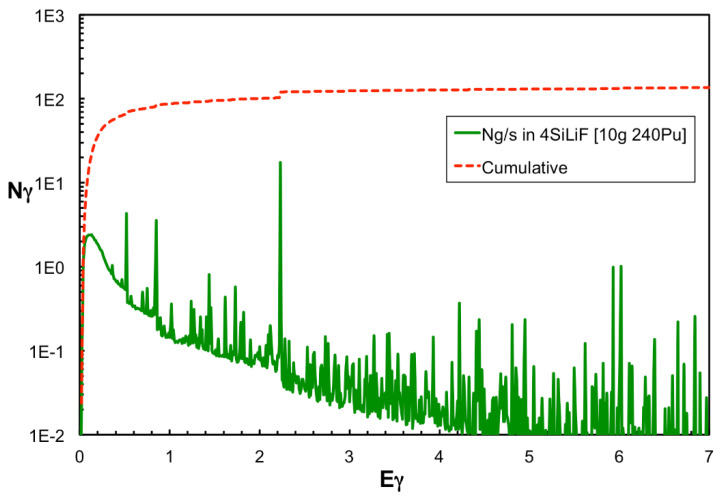
The simulated spectrum of the gamma rays impinging on the four SiLiF detectors (green line) and the corresponding cumulative distribution (red dotted line).

A Monte Carlo simulation of this setup was done by means of the MCNP code, which produced the impinging flux onto the detectors as shown in Figure 9. By combining this flux with the simulated detector efficiency we obtained 4.9 counts/s to be expected in the set of four detectors (i.e., ≈1.2 counts/s/detector), very close to the value from the rough calculation above. The same simulation also produced the spectrum of the gamma rays impinging on the four SiLiF detectors, which is shown in Figure 25 along with the cumulative one. The most prominent peak, as expected, is around 2.2 MeV due to the H(n,γ)D reaction in polyethylene. Simple integrations of this spectrum show that the expected rate of gamma rays between 1 and 2.25 MeV on the four detectors is about 30 counts/s, and that above 2.25 MeV one expects a rate of about 14 counts/s, which is well discriminated from neutrons by the SiLiF.

The expected neutron counting rate indicates that in a medium-term monitoring a set of four SiLiF placed around a drum should in principle be able to detect and monitor the presence of even a lower quantity of ^240^Pu. As also observed in the *top* measurements, the SiLiF detectors can reliably measure at very low neutron counting rates, because of their high neutron selection threshold which cuts off any electronic noise. We remark that in runs of 1–2 h with no radioactive source the SiLiF detectors did not register any neutron count. The only disturbing signals could come from a very intense high-energy gamma ray flux several orders of magnitude higher than the neutron flux, as shown in Section 3.2 and Figure 14. This implies that, under the assumption of a similar flux of neutrons and high-energy gamma rays, the sensitivity to neutrons is at least of the order of 10^−3^–10^−4^. For instance, being the expectation about 1 count/s on each detector, the difference between 90 g and 100 g of ^240^Pu would be seen at one standard deviation in just two minutes, whereas 10 g of ^240^Pu would produce ≈100 total counts in one detector in less than 20 min.

In the MICADO perspective the drums will be first characterized with several methods based on active and passive neutron and gamma ray measurements, then the monitoring detectors will be used to check the longer-term stability of their radiological behavior. Possible counting asymmetries between the four detectors would signal an asymmetry in the fissile distribution, or, should this occur afterwards, a change in the internal structure of the radwaste package. Obviously, for a more precise evaluation of the sensitivity one should take into account also the background from the neighboring drums.

One last point concerns the possible radiation damage of silicon in a radiation field. The radiation hardness claimed by the manufacturer of the silicon is up to 10^13^ n/cm^2^. It is known that the main damage comes from fast neutrons which can dislocate silicon atoms thus creating defects in the semiconductor lattice, with a realistic energy threshold for dislocation in silicon of 34 eV [25], roughly corresponding to the deposited energy of a recoil from a neutron with average energy of about 400 eV. However, even considering the total neutron flux on the detectors, that is about 11 n/cm^2^/s from the simulation, a life longer than many hundreds of years is to be foreseen under these conditions. Indeed, as observed in reference [26], similar detectors were exposed to a fast neutron fluence of 3 × 10^10^/cm^2^ without appreciable changes in their characteristics.

**Table 2:** Calculation of the rate of fission neutrons produced by 100 g of ^240^Pu (the relevant nuclear data were taken from [27]).

Fissioning Species	^240^Pu
T1/2 [y]	6561
T1/2 [s]	2.07 × 10^11^
decay constant τ [y]	9466
decay constant τ [s]	2.99 × 10^11^
decay rate [1/s]	3.35 × 10^−12^
fission Branching Ratio	5.70 × 10^−8^
fission rate [1/s]	1.91 × 10^−19^
atomic mass [amu]	240.05
N atoms/gram	2.51 × 10^21^
sample mass [g]	100
N atoms in sample	2.51 × 10^23^
fission rate in sample [1/s]	4.79 × 10^4^
<prompt neutrons>/fission	2.16
neutron rate from sample [1/s]	1.03 × 10^5^

**Table 3:** Rough estimate of the total expected neutron rate on the four silicon detectors, considering a uniform output flux from the radioactive waste package (RWP) cylinder surface, a 10% neutron absorption inside the drum and fully thermalized neutrons reaching the ^6^LiF converter.

Radwaste Package Matrix	Inox/CH2 67/33%
RWP radius [cm]	28.5
RWP extended radius [cm]	38.5
RWP height [cm]	86
RWP extended side area [cm^2^]	20,804
RWP top + bottom area [cm^2^]	9313
RWP total exit area	30,117
SiLiF active area [cm^2^]	9
number of SiLiF units	4
rough geometrical efficiency	1.20 × 10^−3^
thermal neutron detection efficiency	4%
total neutron counting efficiency	4.8 × 10^−5^
n absorption factor (guess)	0.1
counts/s in 4 SiLiF from sample	4.5

## 5. Conclusions

The described tests, measurements and simulations have allowed us to prove the suitability of the SiLiF technology for neutron detection. The set of 32 detectors we built proved to have a uniform behavior, apart from an unforeseen systematic shift of the detection efficiency due to the converter substrate, which was investigated and fully understood. We can conclude that SiLiF is quite a promising candidate for future applications for low and intermediate level radioactive waste monitoring, and is going to be tested soon in a real environment within the MICADO project.

## Figures and Tables

**Table 1:** Comparison of the measured neutron counting rates to the expectation from simulations.

	Measured Counts/s	Simulated Counts/s
*front*	31.32 ± 0.16	≈32
*inside*	39.78 ± 0.18	≈54

## Data Availability

The data are in the document.
